# Diphtheria and Membranous Croup

**Published:** 1895-02

**Authors:** L. W. Hollis

**Affiliations:** Abilene, Texas


### Correspondence.

### Diphtheria and Membranous Croup.

*Editor Texas Medical Journal:*

For some time I have been reading a discussion in the *Dallas News* and *Fort Worth Gazette*, between Drs. Bealle and McKnight, of Fort Worth, relative to the difference between diphtheria and membranous croup. I am young in the profession, having only twelve years experience, and do not contribute this article to the JOURNAL for the purpose of controversy, far from it. But one of the principal reasons that I ask for space in one of our Texas medical journals is, to ask the profession of Texas to report the cases of membranous croup that each has had, and note how many cases of so-called membranous croup have, like diphtheria, proved contagious. All authority that I have searched claims them two separate and distinct diseases. And when Dr. Bealle (whom I know well, and admire) spoke out, through the columns of the *Dallas News*, saying that they were the same disease, it startled me. I could hardly believe that he said it, so I wrote him at once, and his answer verified the statement, and further, he referred me to the *New York Record* of September 24, 1894, saying it was a *settled fact.* In a few days I find a letter to him, from Cyrus Edson, M. D., Health Physician of New York City, saying that they were identical, and was the result of bacteriological work. Saying further, that because a case of membranous croup does not spread to others, is no reason why it is not contagious, for we see cases of undoubted diphtheria in families, where none others were infected, though subjected to it. I admit all this seems strong evidence, but it is certainly hard for one to concur in such belief when his own experience, though very limited, points directly opposite; and further, when such men as DaCosta, Loomis, Burnette, and others, claim them as two separate and distinct diseases.

Dr. McKnight now speaks in defense of the duality of the two diseases, and gives his reasons as follows: 1st. That diphtheria is highly contagious, and membranous croup is not. 2d. Diphtheria is highly infectious, membranous croup is not infectious. 3d. Diphtheria is inoculable, membranous croup is not inoculable. 4th. In diphtheria, the membrane primarily invades the tonsils, uvula and pharynx, while in membranous croup it primarily invades the larynx. 5th. In diphtheria, there is usually serious systemic disturbance, while in membranous croup there is very little systemic disturbance. 6th. In diphtheria, the glands of the neck are secondarily invaded, in membranous croup they are never involved. 7th. Diphtheria is usually epidemic, membranous croup is never epidemic, but always sporadic. 8th. In diphtheria, paralysis is a common sequella, in membranous croup this is never found. 9th. In diphtheria, Klebs-Loeffler bacillus is found, in membranous croup it is never found. 10th. In diphtheria, albumen is nearly always found in the urine, in membranous croup it is never found.

With the above facts before us, it is hard to believe them one and the same disease. And further, Dr. McKnight also has a letter from New York, written by the celebrated Dr. R. C. M. Page, a professor in the Polyclinic of the City of New York, relative to the details of the two diseases. He says, "I believe in the duality of membranous croup and diphtheria. Both produce strangulation if they invade the larynx, but the pathology of these two diseases is absolutely different." Saying further, that "it is as absurd to state that a child who has been choked to death by a rope around his neck has died of diphtheria, as it is to state that a child who has died of idiopathic membranous croup has died of diphtheria."

This likewise is some evidence that they are separate and distinct diseases. I warrant the assertion, that not a doctor in the State of Texas, except some in the larger cities of the State, can call to mind a single case of membranous croup that, like diphtheria, has proved contagious. I have had nine cases of membranous croup in the last twelve years, and in not a single case have I found it either contagious or infectious, and at the same time everything was favorable for the spread of the disease, if in the least contagious or infectious. For, believing then, as I undoubtedly do now, that it was not contagious or infectious, I did not prohibit the rest of the children of the family from the bedside of the little sufferer. No, nor did I even prohibit them from kissing the dying lips of the child. The fatality of this disease is not in question, but I wish to say that diphtheria is in no wise more fatal than membranous croup, and as a remedy for it, in my opinion, there is none save the knife (in tracheotomy), and that must not be postponed too long, or it will do no good.

Of the nine cases coming under my observation and care, seven died. Six died without the operation, one died during the operation, and two were snatched from death by the timely use of the knife.

I wish here to report the last case that came into my hands, that is of very recent date.

I was called, on the night of the 22d day of October, to see a three years old child of one of our towsmen, Mr. McD., who had, as he thought, ordinary croup, or spasmodic croup. When I arrived, I found her with that peculiar cough you always find in croup. Not attaching much importance to it, thinking it spasmodic croup, and would soon subside, I gave her Sy. Ipecac and Bro. Amo., and directing them how to administer it, left for home, promising to call by next morning. In making my morning calls, I found her, to my disappointment, no better. I at once suspected a more serious trouble, "membranous croup," so I made a thorough examination of the throat, but found nothing but a congested, dry condition of it. I then ordered the inhalation of steam, with no good results perceptible. Knowing now that I had undoubtedly a case of membranous croup to contend with, although not having yet seen the membrane, I at once put her on Mur. Tr. Iron and Potass. Chlo. every two hours, and at the same time used a mop as far down her throat as possible, immersed in a mixture of Borac. Acid ʒi, Tanno. Glyc. ʒij, F. E. Hydrastis Cand. 25 ℥i, hoping that it might have some influence if in contact with the invading membrane; but if either did any good, I failed to see it. In fact, nothing that I did made any impression upon it. Later in the evening, I could plainly see the membrane. The breathing became worse, and I knew now, as I expected from the beginning, that tracheotomy was the only hope for recovery. So I at once informed the parents, making all necessary arrangements to operate when the emergency came. At 10:30 o'clock that night it was plainly evident that the time of action was at hand. So I had my friend, Dr. W. W. Wallace sent for, to assist me. He came, and found matters as I saw them, and we at once sent for our worthy and reliable druggist, Mr. Sam Stenson, who administered the chloroform for us, and with the assistance of Dr. Wallace, I opened the trachea just beneath the cricoid cartilage. We had no hemorrhage, nor any trouble in introducing the tube, and after a few minutes the breathing became easy, and she lapsed into a quiet sleep, remaining so for several hours. In this instance, the knife undoubtedly saved her from death, and that in a few hours. She progressed splendidly, and on the tenth day the throat and trachea trouble had ceased. She could run about the house and yard all day, with the tube tightly corked, breathing with no difficulty through the larynx. Thinking that the time had come to remove the tube, we did so, applying two or three layers of gauze around the neck, covering wound. She went along nicely, breathing through the proper channel, until late in the evening, when the breathing became wonderfully embarrassed, so much so that the reintroduction of the tube became urgently necessary. I put it back, and let her wear it for two weeks longer, and took it out again, removing it early in the morning. She did nicely again, until 10 o'clock at night. As soon as she went to sleep, the breathing became hard, and the family sent for me at once. I found her breathing very difficult when asleep, in fact, she could not sleep. I had Dr. Wallace sent for. We both watched her for two hours or more. The breathing became little better, seemingly, and Dr. Wallace went home, both of us thinking possibly it might subside, knowing that it was due to the want of harmony of the respiratory muscles. He had not left me long until she awoke from a short sleep, fighting for breath. I saw at a glance that something had to be done quickly, or she would expire. I at once tried to introduce a small tube, but failed, then a smaller, and failed. The opening had closed almost entirely—so much so that you could scarcely introduce a curved aneurism needle. I did use the needle, however, but not until all life seemingly had gone. She was in a manner dead. I separated the trachea wound with the needle, applied my mouth to the wound, and inflated the lungs three or four times, and succeeded in introducing a small rubber tube. The breathing came back in sighs, the livid face began to show signs of returning life, and in two hours she was resting well, and slept the balance of the night, waking up in the morning as bright as a lark. I put in a small silver tube the next morning, stopping it up well with a cork, and letting her breathe through the larynx. She is doing nicely, with no more trouble. I shall let her wear the tube until she can sleep well with it tightly corked, before removing it again, feeling certain that the trouble is due to the want of harmony of the respiratory muscles of the larynx and glottis.

I have diverged somewhat from the subject in reporting this case, but hope you will pardon me, for in reporting this case, besides its features of interest, I wish to show that it certainly could not have been contagious or infectious, as Mr. McD. had four other children in the house—yes, in the same room,—and none were infected, though it seems to me that everything was very favorable for them to have been infected, if infectious.

In the other eight cases, it was the same as regards the chances of infection. While this is a report of only nine cases, it does seem to me that it should have some weight as regards its contagiousness and infection. Let the doctors of the State report their cases of membranous croup, and see how many cases are found to be contagious or infectious.

As to the question being settled, I am not satisfied, and desire more evidence than Dr. Bealle and Dr. Edson's opinion on the matter. One thing is very certain to my mind, and that is, if they are one and the same disease, our definition of diphtheria is absolutely wrong.

Fraternally yours,

Abilene, Texas. L. W. HOLLIS, M. D.

